# Case Report: Diet-associated iodine-deficiency goitrous hypothyroidism in a dog: a longitudinal clinical, endocrine, and ultrasonographic evaluation

**DOI:** 10.3389/fvets.2026.1761152

**Published:** 2026-08-13

**Authors:** Sohee Lim, Sang-June Sohn, Yeon-Jung Hong

**Affiliations:** Western Referral Animal Medical Center, Seoul, Republic of Korea

**Keywords:** bilateral goiter, canine hypothyroidism, goitrous hypothyroidism, home-prepared diets, iodine deficiency, serum inorganic iodine, thyroid gland enlargement

## Abstract

Iodine-deficiency hypothyroidism is well recognized in humans, but is considered rare in dogs, largely because commercial canine diets typically meet the established iodine requirements. Herein, we describe a dog that developed primary hypothyroidism with bilateral goiter attributable to dietary iodine deficiency. A 9-year-old spayed female Chihuahua fed a non-standardized home-prepared diet without vitamin and mineral supplementation presented with mild weight gain and bilaterally enlarged thyroid lobes, but without the classic clinical signs of hypothyroidism. Basal thyroid hormone testing revealed markedly decreased total and free thyroxine concentrations with pronounced elevation of thyroid-stimulating hormone levels, consistent with primary hypothyroidism. Ultrasonography revealed symmetric thyroid enlargement with preserved architecture and mild diffuse hypoechogenicity. Fine-needle aspiration excluded neoplasia, and the serum inorganic iodine concentration was severely decreased. The dog’s diet was changed to a commercially prepared, iodine-supplemented formula without thyroid hormone replacement. Over the following 2 months, serum thyroid hormone concentrations normalized, serum iodine concentrations returned to reference values, and ultrasonographic abnormalities improved markedly, with a substantial reduction in thyroid size and restoration of homogeneous echogenicity. This case highlights the potential for iodine-deficiency hypothyroidism to occur in dogs consuming unbalanced home-prepared diets and demonstrates the reversibility of functional abnormalities and marked ultrasonographic improvement following restoration of adequate dietary iodine intake.

## 1 Introduction

Hypothyroidism is one of the most frequently diagnosed endocrine disorders in canine clinical practice, despite an overall population prevalence of only 0.2–0.8% (1). Most affected dogs have primary hypothyroidism resulting from intrinsic thyroid gland dysfunction (2). The predominant etiologies, lymphocytic thyroiditis and idiopathic follicular atrophy, are characterized by a progressive and largely irreversible loss of functional thyroid tissue and a corresponding reduction in glandular volume. Less common causes, including congenital defects, drug-induced dysfunction, neoplastic destruction, and iodine deficiency, have also been described, but occur far less frequently in dogs than in humans (1–3).

In humans, inadequate iodine intake remains the leading cause of goiter and hypothyroidism, particularly in populations that do not routinely consume iodized salts (4, 5). To date, only two veterinary reports have described diet-associated iodine deficiency in dogs: an early account from 1986 that included limited diagnostic evaluation (6), and a more recent, comprehensive case that incorporated ultrasonographic, hormonal, urinary iodine, and scintigraphic assessment in a dog fed an unbalanced home-prepared diet for 1 year (3). A recent report demonstrated that iodine deficiency can cause profound hypothyroidism accompanied by bilateral thyroid enlargement, potentially mimicking neoplastic or immune-mediated thyroid disease. Despite this, the clinical course, diagnostic value of serum iodine measurement, and the degree of structural reversibility of the thyroid gland following iodine repletion remain insufficiently characterized in veterinary patients.

This case report describes a dog with diet-associated iodine-deficiency goitrous hypothyroidism, emphasizing the diagnostic integration of serum inorganic iodine concentrations, ultrasonography, cytology, and serial thyroid hormone monitoring. A longitudinal clinical course following iodine repletion is presented, providing additional insights into the functional recovery and morphologic changes observed following dietary iodine repletion in dogs.

## 2 Case description

A 9-year-old spayed female Chihuahua was routinely managed by a primary care veterinarian and periodically evaluated by the Internal Medicine service at our private referral veterinary hospital for chronic pancreatitis and biliary sludge. For approximately 5 months, the owner had been feeding a home-prepared diet consisting of white rice, brown rice, black rice, skinless chicken breast, cabbage, and pumpkin. According to the owner, the total daily amount was based on the feeding recommendation for a commercial gastrointestinal diet; however, the exact quantities and proportions of individual ingredients could not be retrospectively determined. Digestive enzyme and probiotic supplementation had previously been prescribed by the primary care veterinarian for management of chronic pancreatitis and biliary sludge. Routine hematologic and serum biochemical testing performed by the primary care veterinarian revealed unremarkable hematologic findings. Serum biochemical abnormalities included markedly increased alkaline phosphatase activity [1,552 U/L; reference interval (RI), 13–82] and mild hyperglycemia (134 mg/dL; RI, 70–118). Serum cholesterol concentration (182 mg/dL; RI, 135–345) was within the reference interval, whereas serum triglycerides were not measured. No history of exogenous glucocorticoid administration was reported.

Later that day, during the scheduled evaluation at our referral hospital, the dog was bright, alert, and active, with a good appetite. Body weight had increased from 4.48 to 4.80 kg over approximately 4 months. No lethargy, dermatologic abnormalities, neuromuscular weakness, vomiting, diarrhea, or other gastrointestinal signs were reported. Body condition score was 6/9 and muscle condition score was 3/3. Rectal temperature was 39.6 °C, heart rate was 180 beats/min, respiratory rate was 36 breaths/min, and systolic blood pressure was 170 mmHg. Abdominal ultrasonography confirmed chronic pancreatitis and biliary sludge without other clinically significant abnormalities, and echocardiography was unremarkable.

**Figure 1:**
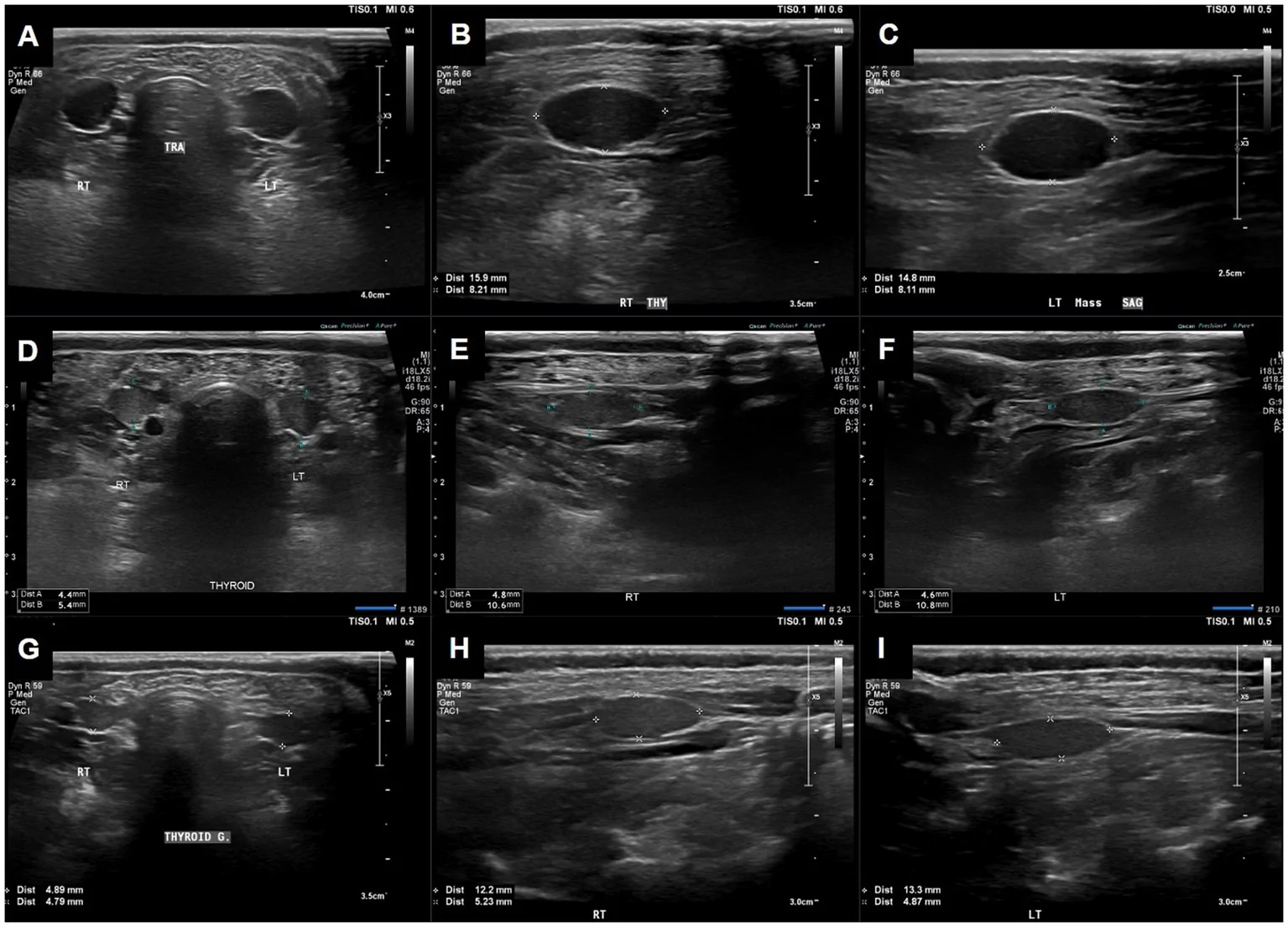
Serial ultrasonographic evaluation of the thyroid glands in a dog with diet-associated iodine-deficiency goitrous hypothyroidism. **(A–C)** Baseline ultrasonography demonstrating bilaterally symmetric enlargement of the thyroid lobes. The thyroid parenchyma was mildly hypoechoic and homogeneous with smooth, well-defined margins and no evidence of nodularity or local invasion. **(D–F)** Three weeks after dietary correction, both thyroid lobes showed a marked reduction in size with restoration of homogeneous echogenicity. **(G–I)** Two months after dietary correction, the thyroid lobes remained symmetric with homogeneous echogenicity, smooth margins, and no focal lesions.

Serum thyroid hormone results obtained by the primary care veterinarian became available shortly after the scheduled evaluation, revealing total T4 < 0.5 μg/dL, free T4 < 0.3 ng/dL, and a markedly increased TSH concentration (2.19 ng/mL; RI, 0.05–0.42). The dog returned to our hospital four days later for further endocrine evaluation. At the endocrine evaluation, cervical ultrasonography demonstrated bilaterally symmetric enlargement of the thyroid lobes (right: 15.9 × 8.2 mm; left: 14.8 × 8.1 mm), with smooth margins and mild diffuse hypoechogenicity (see Figure 1). Based on these findings and the abnormal thyroid hormone results, ultrasound-guided fine-needle aspiration, a comprehensive IDEXX thyroid panel, and serum inorganic iodine measurement were recommended. Three days later, the owner elected to proceed with the recommended diagnostic tests. Ultrasound-guided fine-needle aspiration cytology yielded a low-cellularity sample containing scattered, well-differentiated follicular epithelial cells with minimal atypia and no cytologic evidence of neoplasia. A separate serum sample was submitted for an IDEXX thyroid panel, which confirmed markedly decreased total T4 (1 nmol/L; RI, 9–52), free T4 (3 pmol/L; RI, 6–42), and total T3 (0.4 nmol/L; RI, 0.5–1.4), along with an increased cTSH (2.31 ng/mL; RI, 0–0.58). The patient tested negative for thyroglobulin autoantibodies (TGAA). Serum inorganic iodine concentration was <25 ng/mL (laboratory-provided livestock RI, 50–100). Although canine reference intervals have not been established, this concentration was substantially lower than baseline serum iodine concentrations reported in healthy experimental dogs (7). Taken together with the endocrine abnormalities, symmetric thyroid enlargement, negative TGAA results, absence of cytologic evidence of neoplasia, and the dietary history, these findings supported a diagnosis of iodine-deficiency-induced goitrous hypothyroidism.

The home-prepared diet was replaced with a commercial low-fat diet containing 1.7 mg iodine/kg (Hill’s Prescription Diet i/d Low Fat). According to the owner, digestive enzyme supplementation was continued after the dietary change. The daily energy requirement (DER) was estimated using maintenance energy coefficients (*K* = 95–130 kcal/kg^0.75^/day). Based on a body weight of 4.8 kg (metabolic body weight, 3.24 kg^0.75^), the estimated DER ranged from 308 to 422 kcal/day. Using the manufacturer’s reported metabolizable energy density (3,311 kcal/kg as fed), the estimated daily iodine intake after dietary change was 0.048–0.065 mg/kg^0.75^/day, which was above the FEDIAF 2025 minimum recommended iodine intake for adult dogs (0.03 mg/kg^0.75^/day) (21). No thyroid hormone supplementation was administered.

At the three-week recheck, the dog remained clinically normal with normal fecal characteristics, and the serum total T4 concentration had increased to 1.8 μg/dL. Ultrasonography demonstrated a marked reduction in thyroid size (approximately 10.6–10.8 × 4.6–4.8 mm) and a return to homogeneous parenchymal echogenicity. At 2 months, repeat IDEXX testing showed total T4 of 1.4 μg/dL (RI, 1.0–4.0), free T4 of 15.4 pmol/L (RI, 7.7–47.6), and normalization of cTSH (0.09 ng/mL; RI, 0.05–0.42), with serum inorganic iodine restored to 71 ng/mL. Thyroid lobes remained symmetric with normal echotexture and no nodularity. At 3 months, the total T4 level remained within the reference range (1.9 μg/dL). Serial serum thyroid hormone and serum inorganic iodine measurements are summarized in Table 1.

**Table 1:** Serial serum thyroid hormone concentrations and serum inorganic iodine measurements.

Parameter	Pre-referral^a^	Baseline^b^	3 weeks	2 months^b^	3 months
Total T4	<0.5 μg/dL	1 nmol/L (RI 9–52)	1.8 μg/dL	1.4 μg/dL (RI 1.0–4.0)	1.9 μg/dL
Free T4	<0.3 ng/dL	3 pmol/L (RI 6–42)	—	15.4 pmol/L (RI 7.7–47.6 pmol/L)	—
TSH	2.19 ng/mL (RI 0.05–0.42)	2.31 ng/mL (RI 0–0.58)	—	0.09 ng/mL (RI 0.05–0.42)	—
Total T3	—	0.4 nmol/L (RI 0.5–1.4)	—	—	—
TGAA	—	Negative	—	—	—
Serum inorganic iodine	—	<25 ng/mL^c^	—	71 ng/mL^c^	—

a Pre-referral thyroid hormone measurements were performed by the referring veterinarian using a different assay platform; therefore, units and reference intervals are not directly comparable with those of the IDEXX thyroid panel.

b Baseline and 2-month thyroid hormone measurements were performed using the IDEXX thyroid panel. Units and reference intervals are presented as reported by the laboratory.

c Laboratory-provided livestock reference interval: 50–100 ng/mL.

## 3 Discussion

This case represents a clinically documented example of diet-associated iodine-deficiency goitrous hypothyroidism, in which biochemical, imaging, and nutritional findings converge to demonstrate both the development and reversibility of thyroid dysfunction. Although iodine deficiency is the leading global cause of goiter and hypothyroidism in humans (5), it remains uncommon in dogs due to routine consumption of commercially formulated diets that meet established iodine requirements. However, home-prepared diets provided without appropriate vitamin and mineral supplementation may result in imbalances of multiple nutrients, including deficiencies of micronutrients such as iodine, which are required in only small quantities and may therefore be overlooked in clinical practice. Although regional variation in dietary iodine availability may influence iodine intake, the prolonged consumption of such an unsupplemented home-prepared diet was considered the more likely contributor to iodine deficiency in this case.

Inadequate iodine intake limits thyroid hormone synthesis, resulting in reduced circulating thyroxine concentrations and loss of negative feedback on the hypothalamic–pituitary axis. This leads to sustained TSH stimulation, which in turn induces follicular hypertrophy and hyperplasia, ultimately producing goitrous enlargement of the thyroid gland (4, 5). Experimental studies in dogs have demonstrated that the thyroid gland undergoes dynamic structural and functional adaptation under conditions of restricted iodine intake, including increased iodine trapping, follicular hyperplasia, and subsequent remodeling (8). The findings in the present case closely parallel these mechanisms, with marked TSH elevation, reduced thyroid hormone concentrations, and bilaterally enlarged thyroid glands observed at presentation. Importantly, both endocrine and structural abnormalities improved following dietary correction, supporting the concept that iodine deficiency induces a functional and potentially reversible thyroid state rather than irreversible glandular destruction. This distinction is clinically relevant because most naturally occurring forms of canine hypothyroidism are associated with irreversible thyroid injury (2). In contrast, the preservation of glandular architecture and the rapid normalization of thyroid function observed in this case are more consistent with a hyperplastic process driven by chronic TSH stimulation. Although negative thyroglobulin autoantibody results do not definitively exclude autoimmune thyroiditis (9), the combination of symmetric enlargement, absence of cytologic atypia, and rapid response to iodine repletion makes destructive or neoplastic etiologies unlikely.

A key feature that strengthens the interpretation of this case is the markedly decreased serum iodine concentration at diagnosis and its normalization following dietary correction. Although urinary iodine concentration (UIC) is widely used to assess the iodine status in human population studies (5), its utility in individual clinical patients is limited by substantial diurnal and physiological variations (10, 11). In veterinary medicine, the practical challenges of timed or 24-h urine collection further restrict the applicability of UIC. In contrast, serum iodine measurement reflects integrated intestinal absorption, circulating distribution, thyroidal uptake, and renal elimination, and demonstrates greater intra-individual stability (12–14). In addition to studies involving iodine excess, serum iodine concentrations have also been evaluated under iodine-deficient conditions, including severe depletion during low-iodine dietary interventions and mild deficiency states in humans (14, 15). In this case, the normalization of serum iodine levels closely paralleled the recovery of thyroid hormone concentrations following dietary correction, supporting the potential clinical value of serum inorganic iodine as an adjunct biomarker. Although canine reference intervals have not been established, a baseline serum iodine concentration of 145.9 ± 64.3 ng/mL has been reported in healthy experimental dogs (7), providing a possible comparative value for interpretation. Nevertheless, the absence of species-specific reference intervals and validated diagnostic thresholds in dogs currently limits the broader interpretative utility of serum iodine measurement.

Another important aspect of this report is the longitudinal ultrasonographic evaluation of the thyroid gland. At presentation, ultrasonography revealed diffuse, symmetric enlargement with mild hypoechogenicity and preserved architecture. Diffuse hypoechogenicity has been described in several non-neoplastic thyroid disorders in dogs, including autoimmune thyroiditis and other primary thyroid dysfunctions (16). In human diffuse thyroid diseases, increased follicular epithelial cellularity and reduced colloid content, key histological features of hyperplastic remodeling, are recognized contributors to hypoechogenicity (17). The marked reduction in thyroid size together with restoration of homogeneous echogenicity and parenchymal architecture observed after dietary correction provides *in vivo* evidence of structural recovery, which is rarely documented in veterinary patients. Unlike the case reported by Yun et al. (3), in which thyroid enlargement persisted despite treatment, the present case demonstrated marked improvement in thyroid morphology following dietary correction.

To further evaluate the likelihood of insufficient iodine intake, a semi-quantitative dietary assessment was performed based on the reported diet composition. Daily intake was approximated using reasonable assumptions derived from the patient’s body weight and expected energy requirements for a small-breed dog with stable to mildly increased body weight. Using publicly available food composition data (U.S. Department of Agriculture FoodData Central) (18) and established dietary assessment approaches (18), the estimated iodine intake was approximately 0.0006–0.0015 mg/kg^0.75^/day across multiple plausible intake scenarios. This estimated intake was markedly below the FEDIAF 2025 minimum recommended iodine intake for adult dogs (0.03 mg/kg^0.75^/day), supporting the likelihood of severe dietary iodine insufficiency. These findings should be interpreted with caution because precise quantification was limited by incomplete dietary records and variability in iodine content of individual food items.

Interestingly, despite marked biochemical hypothyroidism, the dog exhibited minimal clinical signs. Several physiological mechanisms may have contributed to this discrepancy. During iodine deficiency, intrathyroidal autoregulation favors the synthesis and secretion of T3 relative to T4, thereby conserving iodine while maintaining production of the more biologically active thyroid hormone (5, 19). In addition, plasma thyroid hormone-binding proteins provide a circulating hormone reservoir and buffer hormone delivery to tissues, while peripheral conversion of T4 to T3 and tissue-specific regulation of intracellular T3 availability may further modulate biological thyroid hormone action (19). These physiological adaptations, together with the gradual and often subtle development of clinical manifestations of canine hypothyroidism (20), may have contributed to the absence of overt clinical signs during the period of dietary iodine deficiency.

This report has some limitations inherent to a single-animal case study. Histopathological confirmation of the thyroid tissue was not available, preventing definitive exclusion of autoimmune or neoplastic disease, although the clinical, cytological, and imaging findings strongly argue against these etiologies. In addition, serum iodine reference intervals for dogs have not yet been established, which limits generalization. Studies with larger sample sizes incorporating standardized iodine biomarkers and longitudinal ultrasonographic follow-ups are needed to better define the diagnostic thresholds and characterize the morphology of nutritional goiters over time. Dietary information was based on the owner report, and the exact quantities and proportions of individual ingredients could not be retrospectively determined, limiting precise estimation of actual nutrient intake.

In summary, this case demonstrates that iodine deficiency should be considered as a reversible cause of primary hypothyroidism in dogs consuming non-standardized home-prepared diets. The combination of decreased serum iodine concentration, characteristic ultrasonographic findings, and rapid normalization following dietary correction provides clinically integrated evidence supporting this diagnosis. As the use of home-prepared diets continues to increase, greater awareness of nutritionally induced endocrine disorders is warranted, along with the incorporation of dietary assessment into routine clinical evaluation.

## 4 Conclusion

This case demonstrates that iodine deficiency from a non-standardized home-prepared diet can cause reversible primary hypothyroidism with bilateral goiter in dogs. Correction of dietary iodine alone resulted in normalization of thyroid hormone concentrations and serum iodine, together with marked ultrasonographic improvement, highlighting the importance of thorough nutritional assessment when evaluating thyroid enlargement or unexplained endocrine abnormalities. Given the growing popularity of home-prepared diets, clinicians should remain vigilant for nutritional deficiencies, particularly micronutrient deficiencies such as iodine deficiency. Additional studies are needed to establish canine-specific iodine reference intervals and to further characterize the ultrasonographic evolution of nutritional goiter.

## Data Availability

The original contributions presented in the study are included in the article/supplementary material, further inquiries can be directed to the corresponding author.
